# Influence of Competing Risks on Estimates of Recurrence Risk and Breast Cancer-specific Mortality in Analyses of the Early Breast Cancer Trialists Collaborative Group

**DOI:** 10.1038/s41598-020-61093-0

**Published:** 2020-03-05

**Authors:** Ramy R. Saleh, Michelle B. Nadler, Alexandra Desnoyers, Danielle L. Rodin, Husam Abdel-Qadir, Eitan Amir

**Affiliations:** 1 Division of Medical Oncology & Hematology, Department of Medicine, Princess Margaret Cancer Centre and the University of Toronto, Toronto, Ontario Canada; 2 Division of Radiation Oncology, Department of Medicine, Princess Margaret Cancer Centre and the University of Toronto, Toronto, Ontario Canada; 3 Department of Medicine, Women’s College Hospital, Toronto, ON Canada; 4 Division of Cardiology, Peter Munk Cardiac Centre and the Ted Rogers Centre for Heart Research, University Health Network, Toronto, ON Canada; 5 Institute for Clinical Evaluative Sciences, Toronto, ON Canada; 6 Institute of Health Policy, Management, and Evaluation, University of Toronto, Toronto, ON Canada

**Keywords:** Breast cancer, Cancer epidemiology, Cancer, Oncology

## Abstract

Early-stage breast cancer (BC) is a curable disease with many patients dying of causes other than BC. The influence of non-BC death and other competing risks on the interpretation of Kaplan-Meier (KM)-based analyses for BC-specific outcomes are unknown. We searched the Oxford University website to identify all meta-analyses published by the Early Breast Cancer Trialists Collaborative Group (EBCTCG) between 2005 and 2018. The potential influence of competing risks was estimated using a validated multivariable linear model that predicts the difference between KM and cumulative incidence function (CIF) on estimates of BC-specific outcomes. The initial search identified 14 EBCTCG papers, 10 (71%) reported data on BC and competing events. Eight (80%) had a relative difference between KM and the competing risk adjusted estimates exceeding 10%. The median relative difference was 28.4% for local-recurrence; 16.8% for distant-recurrence, and 6.7% for BC-specific mortality. There was a 18.9% relative difference between KM and CIF adjusted analyses beyond 10 years. The use of KM-based methods when competing risks are present biases risk estimates in studies of early BC especially for uncommon outcomes such as local recurrence. The use of CIF to calculate BC-specific outcomes may be preferable in this setting.

## Introduction

Early stage breast cancer is a curable disease with many patients dying of causes other than breast cancer^1^. Decisions to recommend adjuvant therapy, whether systemic or radiation, are based typically on the absolute risk of disease recurrence or death from breast cancer. Typically, these risks are estimated using Kaplan–Meier analyses^2,3^ that censor patients without events of interest at the end of the follow-up period^4,5^. Importantly, this method assumes that censored patients have a subsequent event risk that is unchanged in the time following the censoring (‘non-informative censoring’).

A competing risk is defined as an event (e.g. non-breast cancer death) that precludes the occurrence of the primary event of interest (e.g. distant recurrence). Such events do not classify as non-informative since the risk of the studied event changes after the competing risk occurs (i.e. a patient cannot be diagnosed with distant breast cancer recurrence after they die from a stroke). Censoring of competing risks in Kaplan-Meier (KM) analyses will artificially inflate risk estimates, which has been termed immortal time bias^6–8^ or ‘competing-risk bias’. For example, in a cohort of 100 women with breast cancer, of which 2 patients have a distant recurrence every year over 5 years, the risk of distant recurrence is 10% over 5 years in the absence of any competing risks. However, if 10 patients die of non-cancer causes every year for the first 4 years, the same frequency of distant recurrence will result in a KM-based risk estimate of ~14% (see Appendix A for survival table). Increases in the ratio of competing risk relative to the primary event of interest will increase overestimation of the primary outcome risk. A 10% relative difference in estimates of risk of disease-specific events between Kaplan-Meier and cumulative incidence function (CIF) has been identified as methodologically important^3^. This difference is common in the medical literature^9^ with approximately one third of KM analyses overestimating the true risk of an outcome by more than 10%^3^.

Data reported by the Early Breast Cancer Trialists Collaborative Group (EBCTCG) have been instrumental in providing evidence to support treatment planning in early stage breast cancer. However, most analyses performed by EBCTCG have been based on the complement of the KM risk estimate. This will result in biased estimates of breast cancer risk if competing risks are prevalent. Consequently, inaccurate measures of treatment efficacy, especially in the setting of common competing risks, will result. Also, the degree of competing risks is expected to be higher in a routine practice with older patients and multiple comorbidities compared to randomized control trials comprising typically of healthy patients^10^.

Van Walraven and Hawken have derived and validated a method that estimates risk independent of competing risk events from published KM analyses^11,12^. In this article, we applied this method to data reported by EBCTCG to quantify the degree of competing risk bias in pooled analyses of the EBCTCG. We hypothesized that competing risk bias would be greater with longer follow-up and for analyses of less common endpoints (e.g. local recurrence) and that over-estimation of breast cancer-specific events would result in biased estimates of treatment efficacy.

## Methods

### Data source

We searched the Clinical Trial Service Unit and Epidemiological Studies Unit website^13^ at Oxford University to identify all meta-analyses published by the EBCTCG between 2005 and 2018. The search was not restricted to a certain field of research or a specific department. This study was exempt from institutional review board approval since it used publicly available data exclusively.

Studies were included if they contained a Kaplan-Meier (KM) analysis with risk estimates for either breast cancer mortality and/or breast cancer recurrence. Analyses without all-cause death as part of a composite outcome were deemed to be susceptible to competing risk bias unless it was reported explicitly that no competing events occurred. As detailed in individual reports of analyses performed by the EBCTCG, all estimates of recurrence and breast cancer-specific mortality are based on the complement of the Kaplan-Meier curve. As such, estimates of treatment efficacy are also based on this method.

### Data extraction

Two authors (RRS and MN) retrieved the full texts and any supplementary appendices of each included study independently and identified the Kaplan-Meier curves of interest. Discrepancies were resolved by consensus. We selected all Kaplan-Meier curves with breast cancer mortality as an outcome. Additionally, we selected Kaplan-Meier curves for distant recurrence (DR) in studies which analyzed the effects of systemic therapy and curves for local recurrence (LR) in those which analyzed the effects of radiation therapy. The number of events of interest (either breast cancer mortality, distant recurrence, or local recurrence) was extracted from data reported as part of the respective Kaplan-Meier curve for each time frame reported (typically, 0–4 years, 5–9 years and 10–14 years). We extracted the absolute number of non-breast cancer deaths, all deaths, and total recurrences (local, contralateral, and distant) for the same time frames from tables or graphs within the manuscript or supplementary appendices. If not reported, non-breast cancer death was calculated as all deaths minus breast cancer deaths. Competing events were considered as any event (recurrence or death) other than the event of interest as any such event would result in censoring of respective patients in individual trials. For example, the competing risks for LR were any death or any recurrence other than LR. Therefore, the equation used for the Van Walraven and Hawken for LR was (all recurrences + all deaths – LR)/(all recurrences + all deaths).

### Data synthesis and statistical analysis

The potential influence of competing risk bias was estimated using a multivariable linear model that predicts the magnitude to which the KM risk estimates are biased upwards relative to outcome risk measured using the CIF^12^. Briefly, the model used the proportion of all events that are competing events [ie, N(competing events)/N(competing events + outcomes of interest)]. The linear regression model uses two parameters (the KM-based risk estimate *and* the proportion of all events that are competing events) to predict CIF event risk (i.e. the event risk that is not biased by the presence of competing events). The relative difference between Kaplan–Meier and CIF estimates can then calculated. Consistent with prior reporting^3^, a cut point of 10% relative increase was determined as important.

Two authors (RRS and MN) then compared estimates to ensure accuracy. Discrepancies were resolved by consensus. Subsequently, we calculated the relative difference between the risk of each event as estimated by the Kaplan-Meier method and the estimate for the same risk based on CIF (i.e. [(KM risk) − (CIF risk)]/(KM risk)). Data were reported descriptively as the percentage difference between Kaplan-Meier and CIF-based estimates. Differences in the magnitude of treatment effect were reported as the absolute difference, with positive values indicating higher estimates of treatment benefit using Kaplan-Meier analysis and negative values indicating higher estimates with CIF-based analysis. Finally, the formula of the Van Walraven and Hawken model has been described as sensitive to the effects of rounding error. When Kaplan-Meier risks are rounded in the setting of low Kaplan-Meier risk (<0.1) with very low competing events (10%), there can be overcorrection of the CIF estimate, although this should affect only the third decimal digit. When the Kaplan-Meier risks and proportion of competing events met these criteria, they were reported descriptively. No inferential statistical testing was performed.

### Ethical approval and consent to participate

Not applicable for our study as human subjects were not involved.

### Consent to publish

The manuscript has been read and approved by all named authors and that there are no other persons who satisfied the criteria for authorship but are not listed. All authors consent to publish. We further confirm that the order of authors listed in the manuscript has been approved by all of us.

## Results

**Figure 1:**
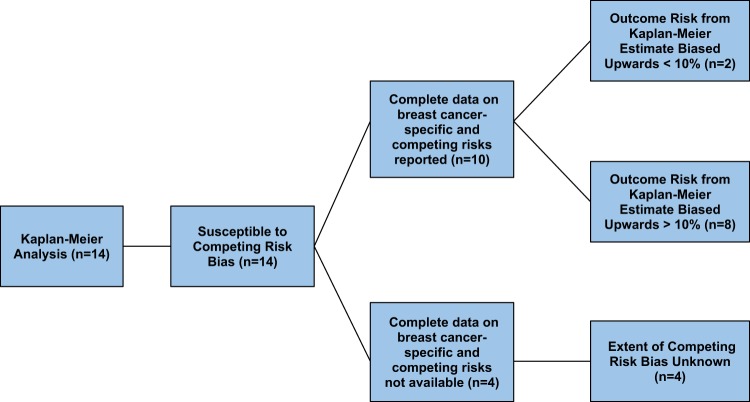
Prevalence of competing risk bias in the 14 published EBCTCG papers with Kaplan-Meier analyses.

The initial search identified 14 analyses published by the EBCTCG^13^ between May 2005 and January 2018 (Appendix B). All 14 studies included Kaplan-Meier-based analyses that were susceptible to competing risk bias (Fig. 1)^14–27^. One study was excluded because its primary outcome was risk of lung cancer death^26^ and three additional studies were excluded because the number of competing events was not reported^14,16,21^. Of the remaining 10 studies, at least one study outcome was all-cause mortality. 4 studies included DR and 2 included LR (Table 1). None of these studies reported the CIF (for outcomes other than all-cause mortality) to account for competing risks.

**Table 1:** Description of the studies searched for possible susceptibility to competing risk bias.

#	Article	Breast Cancer Mortality	Recurrence	Competing variables reported
1	Effects of chemotherapy and hormonal therapy for early breast cancer on recurrence and 15-year survival: an overview of the randomised trials (The Lancet 2005; 365: 1687–1717)	No	No	No
2	Effects of radiotherapy and of differences in the extent of surgery for early breast cancer on local recurrence and on 15-year survival: an overview of the randomised trials (The Lancet 2005; 366: 2087–2106)	Yes	No	Yes
3	Adjuvant chemotherapy in oestrogen-receptor-poor breast cancer: patient-level meta-analysis of randomised trials (The Lancet 2008; 371: 29–40	No	No	No
4	Meta-analysis of breast cancer outcomes in adjuvant trials of aromatase inhibitors versus tamoxifen. Dowsett M. *et al*., (2010), J clin oncol, 28, 509–518	Yes	No	Yes
5	Overview of the Randomized Trials of Radiotherapy in Ductal Carcinoma *In Situ* of the Breast Early EBCTCG J Natl Cancer Inst Monogr. 2010 Oct; 2010(41): 162–177	Yes	Local	Yes
6	Effect of radiotherapy after breast-conserving surgery on 10-year recurrence and 15-year breast cancer death: meta-analysis of individual patient data for 10 801 women in 17 randomised trials EBCTCG. Lancet 2011. Nov 12; 378(9804): 1707–1716	Yes	No	Yes
7	Relevance of breast cancer hormone receptors and other factors to the efficacy of adjuvant tamoxifen: patient-level meta-analysis of randomised trials Early Breast Cancer Trialists’ Collaborative Group (EBCTCG) Lancet 2011. Aug 27; 378(9793): 771–784.	Yes	No	Yes
8	Comparisons between different polychemotherapy regimens for early breast cancer: meta-analyses of long-term outcome among 100 000 women in 123 randomised trials EBCTCG Lancet 2012. Feb 4; 379(9814): 432–444.	No	No	No
9	Effect of radiotherapy after mastectomy and axillary surgery on 10-year recurrence and 20-year breast cancer mortality: meta-analysis of individual patient data for 8135 women in 22 randomised trials EBCTCG. Lancet. 2014 Jun 20; 383(9935): 2127–2135.	Yes	Local	Yes
10	Adjuvant bisphosphonate treatment in early breast cancer: meta-analyses of individual patient data from randomised trials (Lancet 2015; 386: 1353–61)	Yes	Distant	Yes
11	Aromatase inhibitors versus tamoxifen in early breast cancer: patient-level meta-analysis of the randomised trials (Lancet 2015; 386: 1341–52	Yes	Distant	Yes
12	20-year risks of breast-cancer recurrence after stopping endocrine therapy at 5 years. N Engl J Med 2017; 377: 1836–1846	Yes	Distant	Yes
13	Estimating the risks of breast cancer radiotherapy: evidence from modern radiation doses to the lungs and heart and from previous randomized trials. J Clin Oncol 2017; 35: 1641–49	No	No	No
14	Long-term outcomes for neoadjuvant versus adjuvant chemotherapy in early breast cancer: meta-analysis of individual patient data from ten randomised trials. Early Breast Cancer Trialists’ Collaborative Group (EBCTCG) Lancet Oncol 2018. Jan; 19(1): 27–39	Yes	Distant	Yes

Ten of the 14 studies (71%) susceptible to competing risk bias quantified the events of interest and competing events, thereby permitting us to estimate the extent that true outcome risk may have been overestimated by the Kaplan-Meier estimate. The remaining 4 studies (29%) did not report the number of competing events that occurred during the study. As a result, the magnitude of bias in the risk estimates of these studies could not be estimated.

**Figure 2:**
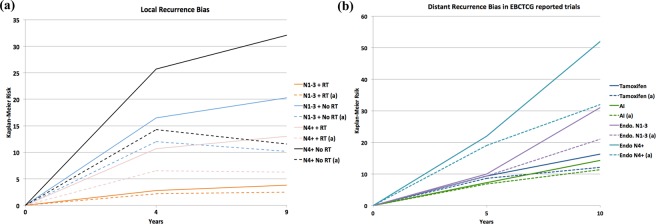
Local (**a**) vs. Distant (**b**) Recurrence Bias Noted in the EBCTCG trials. (RT = radiation therapy, AI = aromatase inhibitor, N = node status, Endo = Endocrine therapy).

**Figure 3:**
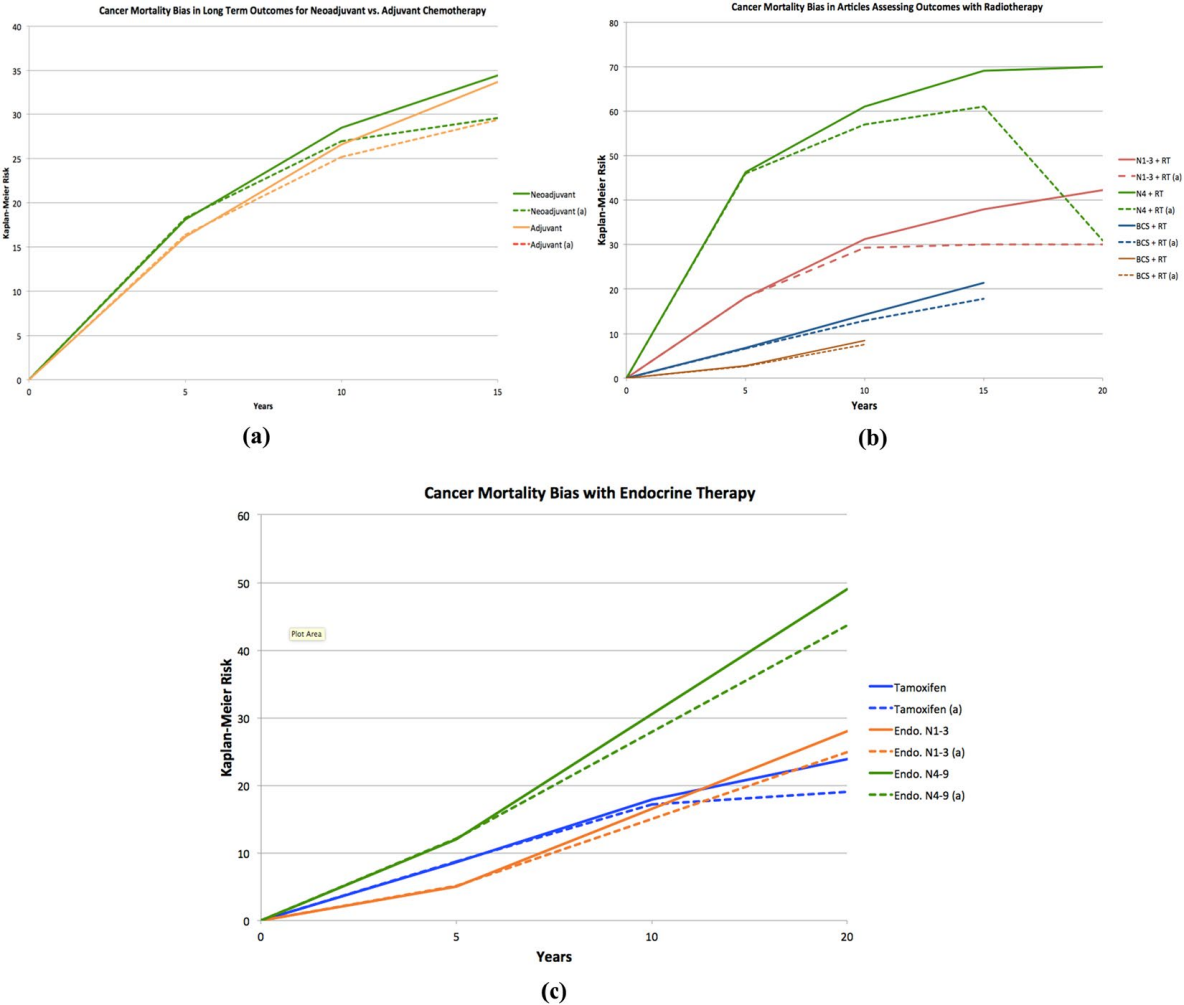
Cancer Mortality Bias in, (**a**) Chemotherapy (**b**) Radiation (**c**) Endocrine therapy, studies published in the EBCTCG papers. (RT = radiation therapy, BCS = breast cancer surgery, N = node status, Endo = Endocrine therapy).

Eight of the ten studies (80%) had a relative difference between the Kaplan-Meier estimate and the competing risk adjusted estimate of more than 10% while 2 of 10 (20%) had Kaplan-Meier percent difference of less than 10% (see Fig. 1). Among all analyses, the average relative difference between the Kaplan-Meier and adjusted estimates was 28.4% for LR (Fig. 2a) 16.8% for DR (Figs. 2b) and 6.7% for breast cancer-specific mortality (Fig. 3a–c). Among all analyses, there was a 2.2% mean relative difference between Kaplan-Meier and adjusted analyses between 0 to 4 years and 18.9% beyond 10 years of follow up (10–14 years or 15–19 years where applicable). Low Kaplan-Meier and competing events rates were observed in 19 of 157 simulations (12%) (See * Appendix B).

Use of Kaplan-Meier and CIF-based analysis did not influence the direction of the treatment effect in most included studies (Appendix C). The absolute effect on treatment could not be calculated in two studies which reported outcomes irrespective of treatment exposure. The larger differences in estimates of treatment effect were noted for the effect of radiation on patients with node positive disease and cancer mortality.

## Discussion

The provision of optimal patient care can be aided by estimates of the absolute risk of events of interest^5^. Accurate estimation of absolute risk permits effective clinical decision-making and risk stratification of patients and forms the basis for estimates of treatment effect^28^.

The International Council for Harmonisation (ICH) has recognized that without a precise understanding of the absolute treatment effect, there is a risk that its magnitude and meaningfulness will be misunderstood. There is a growing awareness of the impact of competing risks in prognostic models especially those predicting disease-specific outcomes such as risk of recurrence^7,9,12^. In an draft addendum to the ICH guideline on statistical principles for clinical trials (ICH E9), the ICH highlighted that there should be measurement of intercurrent events that could lead to ambiguity of the treatment effects and potential misalignment with the trial objectives^29^. This guidance document also recommends that allowances should be made for intercurrent events that occur after randomization and that appropriate methods be used to estimate treatment effects accurately even after an intercurrent event, as well as how to account for the occurrence or non-occurrence of the event itself. Therefore, more broad awareness of different statistical methods for addressing research questions in the presence of competing risks is warranted^30–34^.

The use of conventional survival analysis is the gold standard method for describing the observations in clinical trials. However, when used for prospective modeling of risk to aid in clinical decision-making, the use of conventional methods can be limited in the setting of frequent competing risks. This is especially important for decisions about adjuvant therapy in estrogen receptor-positive early stage breast cancer, a subgroup of breast cancer with frequent competing risks. Data from clinical trials and from analyses of the EBCTCG are used frequently to estimate the risk of recurrence in these patients thereby identifying a group who may benefit most from treatment. However, the use of these data, which are analyzed using conventional survival analysis methods is conditional on the patient surviving a specified period in the future. As the probability of survival is not easy to predict and can be variable between patients this can result in over-estimation of benefit from treatment unless the probability of competing risks is modeled accurately.

Competing risks analysis extends conventional survival analysis. Although the log-rank test and the Cox regression can be adapted easily, they cannot be used to approximate to the CIF because of the presence of competing risks. To overcome this hurdle, specialized methods that target the cumulative incidence function have been developed^35,36^. The Fine and Gray Method^35^ extends the Cox regression to model the CIF and provides modeling techniques for competing risks data. However, the Fine and Gray model has been criticized for its weakness of interpretation. The most marked limitation with Fine and Gray modeling related to model covariates not linking directly to an underlying event rate in the real world^37^.

While there is an extensive literature examining the value of reporting CIF in place of KM^6,7^, when competing risks are observed commonly (such as in early stage breast cancer), it is recommended to report both competing risk and cause-specific models^38^. Unfortunately, despite frequent competing risks, data reported by the EBCTCG do not report competing risks models resulting in uncertainty of risk estimates. Reporting of competing risks modeling for analyses of EBCTCG data is therefore of merit. The median age of breast cancer diagnosis is developed countries is approximately 60 years^1^. As such after 10 years of follow-up, most patients are at a clinically relevant risk of non-breast cancer death. When predicting outcomes such as cancer mortality post-radiation over long periods of time (e.g. 10 years or more), competing risks require careful consideration. Cancer mortality in women with node negative or low nodal burden is low overall, and analyses adjusting for competing risks demonstrate that the absolute overall survival benefit from radiation in this setting may be lower than estimated previously. Breast radiation is known to have adverse cardiovascular outcomes^39^. Exposure of patients at low risk of breast cancer recurrence to radiation is unlikely to result in a substantial treatment benefit yet may increase competing risks such as cardiovascular disease. In such a situation Kaplan-Meier-based analyses can result in substantial immortal time bias and may over-estimate the treatment effect. In those with 1–3 positive nodes and particularly 4 or more positive nodes, the influence of competing risks is less apparent (Fig. 3b) as these patients are at greater risk of distant recurrence and breast cancer mortality.

As expected, an increasing influence of competing risks is observed with longer follow-up. This is explained by data showing increasing proportions of non-breast cancer deaths among patients with early stage breast cancer^1^. The effect of the corrected estimate was lower over time in the chemotherapy trials than in the endocrine therapy trials. This could be explained by the fact that chemotherapy impacts early recurrence whereas endocrine therapy impacts later recurrence. This also provides an important consideration for decisions for extended adjuvant endocrine therapy. Individual trials have reported higher relative benefit form treatment when competing risks are ignored^40,41^.

Our study has limitations. First, we were unable to calculate the corrected Kaplan-Meier in all the studies. This was due to of the design of our model, where it can only be used to predict true event risk if the absolute number of competing events are known and reported. In our case, three studies did not report these events. Second, we were unable to explore the effect of sequential events (e.g. distant recurrence occurring after local recurrence). This may result in an overestimation in the magnitude of competing risk, which therefore inflates the bias attributed to use of the Kaplan-Meier. Finally, the 10% cut-off point for relative increase can be considered contextual; when the absolute risk is low, a 10% relative difference might not be clinically meaningful. Finally, low Kaplan-Meier and competing events rates were observed in approximately 1 in 8 analyses. However, this overcorrection of the CIF estimate, should affect only the third decimal digit and therefore, was not felt to impact substantially on the interpretation of the results.

In conclusion, this study provides overestimation estimates of risk in Kaplan-Meier analyses resulting from failure to address competing risk bias. CIFs may be more appropriate methods to measure outcome risk over time and should be considered, especially for analysis of rare events (e.g. local recurrence) and long-term follow-up.

## Supplementary Information

Supplemental Information.

## Data Availability

Data will be available for 10 years from the date of publication.
