# Supplementary material for: Influence of Competing Risks on Estimates of Recurrence Risk and Breast Cancer-specific Mortality in Analyses of the Early Breast Cancer Trialists Collaborative Group
Source: Sci Rep. 2020 Mar 5;10:4091. doi: 10.1038/s41598-020-61093-0 (PMC7058037; doi:10.1038/s41598-020-61093-0)
Supplement: Supplementary file 1 — Supplemental Information. [file 41598_2020_61093_MOESM1_ESM.docx]

**Appendix A**

**Example illustrating the difference in the estimated cumulative incidence of breast cancer death using the complement of the Kaplan-Meier survival in two settings with different incidence of competing risks.**

| **Kaplan-Meier survival table with no deaths from causes other than cardiovascular disease** | | | | | |
| --- | --- | --- | --- | --- | --- |
| **Year** | **Number at risk** | **Non-cancer death (Censored)** | **Cancer death (Failed)** | **Survival probability estimate** | **Estimated cumulative incidence** |
| **1** | 100 | 0 | 2 | 98% | 2% |
| **2** | 98 | 0 | 2 | 96% | 4% |
| **3** | 96 | 0 | 2 | 94% | 6% |
| **4** | 94 | 0 | 2 | 92% | 8% |
| **5** | 92 | 0 | 2 | 90% | 10% |
| **Kaplan-Meier survival table with 10 non-cancer deaths every year in years 1 through 4** | | | | | |
| **Year** | **Number at risk** | **Non-cancer death (Censored)** | **Cancer death (Failed)** | **Survival probability estimate** | **Estimated event rate** |
| **1** | 100 | 10 | 2 | 98% | 2% |
| **2** | 88 | 10 | 2 | 95.7% | 4.3% |
| **3** | 76 | 10 | 2 | 93.1% | 6.9% |
| **4** | 64 | 10 | 2 | 90% | 10% |
| **5** | 52 | 0 | 2 | 86.1% | 13.9% |

**Appendix B**

**Paper 2: Effects of radiotherapy and of differences in the extent of surgery for early breast cancer on local recurrence and on 15-year survival: an overview of the randomised trials The Lancet 2005; 366: 2087-2106**

**Cancer Mortality Bias**

| **All node negative patients** | | | |
| --- | --- | --- | --- |
| **BCS + RT** | **Reported** | **Adjusted** | **Percentage Difference** |
| **Years 0-4** | 8 | 8.1 | -1.3* |
| **Years 5-9** | 17.4 | 16.8 | 3.4 |
| **Years 10-14** | 26.1 | 24.8 | 5.0 |
|  | | | |
| **BCS no RT** | **Reported** | **Adjusted** | **Percentage Difference** |
| **Years 0-4** | 8.9 | 8.9 | 0 |
| **Years 5-9** | 20.3 | 20.2 | 0.5 |
| **Years 10-14** | 31.2 | 30.1 | 3.5 |

| **All node positive patients** | | | | | |
| --- | --- | --- | --- | --- | --- |
| **BCS + RT** | **Reported** | | **Adjusted** | | **Percentage Difference** |
| **Years 0-4** | 20.9 | | 21.2 | | -1.4* |
| **Years 5-9** | 36.5 | | 35.8 | | 1.9 |
| **Years 10-14** | 47.9 | | 45.8 | | 4.4 |
|  | | | | | |
| **BCS no RT** | **Reported** | **Adjusted** | | **Percentage Difference** | |
| **Years 0-4** | 24.3 | 24.7 | | 1.6 | |
| Years 5-9 | 45.2 | 45.3 | | -0.2* | |
| Years 10-14 | 55 | 50.3 | | 8.5 | |

| **Mastectomy and node negative patients** | | | |
| --- | --- | --- | --- |
| **Mx + RT** | **Reported** | **Adjusted** | **Percentage Difference** |
| **Years 0-4** | 12.5 | 12.5 | 0 |
| **Years 5-9** | 22.3 | 19.8 | 11.2 |
| **Years 10-14** | 31.3 | 26.7 | 14.7 |
|  | | | |
| **Mx no RT** | **Reported** | **Adjusted** | **Percentage Difference** |
| **Years 0-4** | 11.3 | 11.3 | 0 |
| **Years 5-9** | 20.8 | 19.2 | 7.7 |
| **Years 10-14** | 27.7 | 23.3 | 15.9 |

| **Mastectomy and node positive patients** | | | |
| --- | --- | --- | --- |
| **Mx + RT** | **Reported** | **Adjusted** | **Percentage Difference** |
| **Years 0-4** | 32.1 | 32.4 | -0.9* |
| **Years 5-9** | 46.7 | 43.8 | 6.2 |
| **Years 10-14** | 54.7 | 46.5 | 15.0 |
|  | | |  |
| **Mx no RT** | **Reported** | **Corrected** | **Percentage Difference** |
| **Years 0-4** | 34 | 34.4 | -1.2* |
| **Years 5-9** | 50.9 | 49.1 | 3.5 |
| **Years 10-14** | 60.1 | 55.1 | 8.3 |

**Paper 4:** [**Meta-analysis of breast cancer outcomes in adjuvant trials of aromatase inhibitors versus tamoxifen.**](https://www.ctsu.ox.ac.uk/publications/34994) **Dowsett M. et al, (2010), J clin oncol, 28, 509 – 518**

**Cancer Mortality Bias**

| **Cohort 1 vs 2** | **Reported** | **Adjusted** | **Percentage Difference** |
| --- | --- | --- | --- |
| **Tamoxifen in Cohort 1** | | | |
| **Years 0-4** | 5.9 | 5.8 | 1.7 |
| **Years 5-9** | 10.5 | 9.9 | 5.7 |
|  | | | |
| **AI in Cohort 1** | | | |
| **Years 0-4** | 4.8 | 4.8 | 0 |
| **Years 5-9** | 10 | 9.4 | 6.0 |
|  | | | |
| **Tamoxifen in Cohort 2** | | | |
| **Years 0-4** | 2.4 | 2.4 | 0 |
| **Years 5-9** | 7.9 | 7.6 | 3.8 |
|  | | | |
| **AI in Cohort 2** | | | |
| **Years 0-4** | 1.7 | 1.8 | -5.9* |
| **Years 5-9** | 6.3 | 6.1 | 3.2 |

**Paper 5: Overview of the Randomized Trials of Radiotherapy in Ductal Carcinoma In Situ of the Breast EBCTCG J Natl Cancer Inst Monogr. 2010 Oct; 2010(41): 162–177.**

**Local recurrent bias**

| **BCS + RT** | **Reported** | **Adjusted** | **Percentage Difference** |
| --- | --- | --- | --- |
| **Years 0-4** | 7.6 | 7.7 | -1.3* |
| **Years 5-9** | 12.9 | 10.8 | 16.3 |
|  | | | |
| **BCS no RT** | **Reported** | **Adjusted** | **Percentage Difference** |
| **Years 0-4** | 18.1 | 17.5 | 3.3 |
| **Years 5-9** | 28.1 | 23.1 | 17.9 |

**Cancer Mortality Bias**

| **BCS + RT** | **Reported** | **Adjusted** | **Percentage Difference** |
| --- | --- | --- | --- |
| **Years 0-4** | 2.8 | 2.7 | 3.6 |
| **Years 5-9** | 8.4 | 7.5 | 10.7 |
|  | | | |
| **BCS no RT** | **Reported** | **Adjusted** | **Percentage Difference** |
| **Years 0-4** | 2.7 | 2.7 | 0 |
| **Years 5-9** | 8.4 | 7.2 | 14.3 |

**Paper 6:** **Effect of radiotherapy after breast-conserving surgery on 10-year recurrence and 15-year breast cancer death: meta-analysis of individual patient data for 10 801 women in 17 randomised trials EBCTCG Lancet. 2011 Nov 12; 378(9804): 1707–1716**

**Cancer Mortality Bias**

| **RT** | **Reported** | **Adjusted** | **Percentage Difference** |
| --- | --- | --- | --- |
| **Years 0-4** | 6.8 | 6.7 | 1.5 |
| **Years 5-9** | 14.2 | 12.9 | 9.9 |
| **Years 10-14** | 21.4 | 17.9 | 16.4 |
|  | | | |
| **No RT** | **Reported** | **Adjusted** | **Percentage Difference** |
| **Years 0-4** | 7.8 | 7.8 | 0 |
| **Years 5-9** | 17.2 | 16.2 | 5.8 |
| **Years 10-14** | 25.2 | 21.6 | 14.3 |

**Paper 7: Relevance of breast cancer hormone receptors and other factors to the efficacy of adjuvant tamoxifen: patient-level meta-analysis of randomised trials (EBCTCG) Lancet. 2011 Aug 27; 378(9793): 771–784.**

**Cancer Mortality Bias**

| **With Tamoxifen** | **Reported** | **Adjusted** | **Percentage Difference** |
| --- | --- | --- | --- |
| **Years 0-4** | 8.6 | 8.7 | -1.2* |
| **Years 5-9** | 17.9 | 17.2 | 3.9 |
| **Year >= 10** | 23.9 | 19.4 | 18.8 |
|  | | | |
| **Control** | **Reported** | **Adjusted** | **Percentage Difference** |
| **Years 0-4** | 11.9 | 12 | -0.8* |
| **Years 5-9** | 25.1 | 24.1 | 4.0 |
| **Year >= 10** | 33.1 | 27.9 | 15.7 |

**Paper 9: Effect of radiotherapy after mastectomy and axillary surgery on 10-year recurrence and 20-year breast cancer mortality: meta-analysis of individual patient data for 8135 women in 22 randomised trials EBCTCG Lancet. 2014 Jun 20; 383(9935): 2127–2135.**

**Local recurrent bias**

| **Years** | **Reported** | **Adjusted** | **Percentage Difference** |
| --- | --- | --- | --- |
| **N0 with RT** | | | |
| **Years 0-4** | 1.9 | 1.6 | 15.7 |
| **Years 5-9** | 3 | 2.3 | 23.3 |
|  | | | |
| **N0 without RT** | | | |
| **Years 0-4** | 1.2 | 1.0 | 16.7 |
| **Years 5-9** | 1.6 | 1.1 | 31.3 |
|  | | | |
| **N1-3 with RT** | | | |
| **Years 0-4** | 2.8 | 2.2 | 21.4 |
| **Years 5-9** | 3.8 | 2.5 | 34.2 |
|  | | | |
| **N1-3 - without RT** | | | |
| **Years 0-4** | 16.5 | 12.0 | 27.3 |
| **Years 5-9** | 20.3 | 10.2 | 49.8 |
|  | | | |
| **N4+ with RT** | | | |
| **Years 0-4** | 10.7 | 6.5 | 39.3 |
| **Years 5-9** | 13 | 6.3 | 51.5 |
|  | | | |
| **N4+ without RT** | | | |
| **Years 0-4** | 25.7 | 14.3 | 44.4 |
| **Years 5-9** | 32.1 | 11.6 | 63.9 |

**Cancer Mortality Bias**

| **N0 with RT** | **Reported** | **Adjusted** | **Percentage Difference** |  | **N0 without RT** | **Reported** | **Adjusted** | **Percentage Difference** |
| --- | --- | --- | --- | --- | --- | --- | --- | --- |
| Years 0-4 | 11 | 11.1 | -0.9* |  | Years 0-4 | 10.6 | 10.7 | -0.9* |
| Years 5-9 | 18.4 | 16.2 | 12.0 |  | Years 5-9 | 18.3 | 16.6 | 9.3 |
| Years 10-14 | 25.9 | 22.5 | 13.1 |  | Years 10-14 | 23.9 | 20.4 | 14.6 |
| Years 15-19 | 28.8 | 16.3 | 43.4 |  | Years 15-19 | 26.6 | 17.8 | 33.1 |

| **N1-3 with RT** | **Reported** | **Adjusted** | **Percentage Difference** |  | **N1-3 without RT** | **Reported** | **Adjusted** | **Percentage Difference** |
| --- | --- | --- | --- | --- | --- | --- | --- | --- |
| Years 0-4 | 18.1 | 18.1 | 0 |  | Years 0-4 | 22 | 22.2 | -0.9* |
| Years 5-9 | 31.2 | 29.3 | 6.1 |  | Years 5-9 | 36.8 | 34.5 | 6.3 |
| Years 10-14 | 37.9 | 30.1 | 20.6 |  | Years 10-14 | 47 | 42.3 | 10.0 |
| Years 15-19 | 42.3 | 30.1 | 28.8 |  | Years 15-19 | 50 | 35.9 | 28.2 |

| **N4+ with RT** | **Reported** | **Adjusted** | **Percentage Difference** |  | **N4+ without RT** | **Reported** | **Adjusted** | **Percentage Difference** |
| --- | --- | --- | --- | --- | --- | --- | --- | --- |
| Years 0-4 | 46.3 | 46.2 | 0.2 |  | Years 0-4 | 48.4 | 48.2 | 0.4 |
| Years 5-9 | 61.1 | 57.7 | 5.6 |  | Years 5-9 | 67.3 | 65.5 | 2.7 |
| Years 10-14 | 69.3 | 61.2 | 11.7 |  | Years 10-14 | 74.9 | 68.0 | 9.2 |
| Years 15-19 | 70.7 | 31.8 | 55.0 |  | Years 15-19 | 80 | 67.1 | 16.1 |

**Paper 10: Adjuvant bisphosphonate treatment in early breast cancer: meta-analyses of individual patient data from randomised trials (Lancet 2015; 386: 1353-61)**

**Distant recurrence**

| **All Bisphosphonates** | **Reported** | **Adjusted** | **Percentage Difference** |
| --- | --- | --- | --- |
| **Years 0-4** | 13.5 | 12.2 | 9.6 |
| **Years 5-9** | 20.4 | 16.1 | 21.1 |
|  | | | |
| **Control arm** | **Reported** | **Adjusted** | **Percentage Difference** |
| **Years 0-4** | 14.8 | 7.9 | 46.6 |
| **Years 5-9** | 21.8 | 16.7 | 23.4 |
|  | | | |
| **Pre- Bisph.** | **Reported** | **Corrected** | **Percentage Difference** |
| **Years 0-4** | 17.9 | 15.7 | 12.3 |
| **Years 5-9** | 25.6 | 20.3 | 20.7 |
|  | | | |
| **Control arm** | **Reported** | **Adjusted** | **Percentage Difference** |
| **Years 0-4** | 17.7 | 15.5 | 12.4 |
| **Years 5-9** | 24.6 | 18.9 | 23.2 |
|  | | | |
| **Post- Bisph.** | **Reported** | **Adjusted** | **Percentage Difference** |
| **Years 0-4** | 11.1 | 10.2 | 8.1 |
| **Years 5-9** | 17.9 | 14.2 | 20.7 |
|  | | | |
| **Control arm** | **Reported** | **Adjusted** | **Percentage Difference** |
| **Years 0-4** | 13.6 | 12.3 | 9.6 |
| **Years 5-9** | 21.2 | 16.1 | 24.1 |

**Cancer Mortality Bias**

| **All Bisphosphonates** | **Reported** | **Adjusted** | **Percentage Difference** |
| --- | --- | --- | --- |
| **Years 0-4** | 8.9 | 9.0 | -1.1* |
| **Years 5-9** | 16.6 | 16.1 | 3.0 |
|  | | | |
| **Control arm** | **Reported** | **Adjusted** | **Percentage Difference** |
| **Years 0-4** | 9.7 | 9.8 | -1.0* |
| **Years 5-9** | 18.4 | 17.8 | 3.3 |
|  | | | |
| **Pre- Bisph.** | **Reported** | **Adjusted** | **Percentage Difference** |
| **Years 0-4** | 11.8 | 11.9 | -0.8* |
| **Years 5-9** | 20.6 | 20.1 | 2.4 |
|  | | | |
| **Control arm** | **Reported** | **Adjusted** | **Percentage Difference** |
| **Years 0-4** | 12.1 | 12.2 | -0.8* |
| **Years 5-9** | 20.7 | 20.1 | 2.9 |
|  | | | |
| **Post- Bisph.** | **Reported** | **Adjusted** | **Percentage Difference** |
| **Years 0-4** | 7.5 | 7.6 | -1.3* |
| **Years 5-9** | 14.7 | 13.9 | 5.4 |
|  | | | |
| **Control arm** | **Reported** | **Adjusted** | **Percentage Difference** |
| **Years 0-4** | 8.7 | 8.7 | 0 |
| **Years 5-9** | 18 | 17.1 | 5.0 |

**Paper 11: Aromatase inhibitors versus tamoxifen in early breast cancer: patient-level meta-analysis of the randomised trials (Lancet 2015; 386: 1341-52)**

**Distant Recurrence**

| **Cohort 1 - TAM** | **Reported** | **Adjusted** | **Percentage Difference** |
| --- | --- | --- | --- |
| **Years 0-4** | 9.4 | 8.6 | 8.5 |
| **Years 5-9** | 16.3 | 12.1 | 25.8 |
|  | | | |
| **Cohort 1 - AI** | **Reported** | **Adjusted** | **Percentage Difference** |
| **Years 0-4** | 7.3 | 6.8 | 6.8 |
| **Years 5-9** | 14.3 | 11.3 | 21.0 |
|  | | | |
| **Cohort 2 - TAM then AI** | **Reported** | **Adjusted** | **Percentage Difference** |
| **Years 0-4** | 8.4 | 7.8 | 7.1 |
| **Years 5-9** | 11.3 | 9.5 | 15.9 |
|  | | | |
| **Cohort 2 - AI** | **Reported** | **Adjusted** | **Percentage Difference** |
| **Years 0-4** | 7.4 | 6.9 | 6.8 |
| **Years 5-9** | 10.7 | 9.1 | 15.0 |
|  | | | |
| **Cohort 3 - TAM then AI** | **Reported** | **Adjusted** | **Percentage Difference** |
| **Years 2-4** | 7.1 | 6.8 | 4.2 |
| **Years 5-9** | 12.1 | 10.4 | 14.0 |
|  | | | |
| **Cohort 3 - TAM** | **Reported** | **Adjusted** | **Percentage Difference** |
| **Years 2-4** | 8.6 | 8.2 | 4.7 |
| **Years 5-9** | 13.1 | 10.7 | 18.3 |

**Cancer Mortality Bias**

| **Cohort 1 - TAM** | **Reported** | **Adjusted** | **Percentage Difference** |
| --- | --- | --- | --- |
| **Years 0-4** | 5.8 | 5.8 | 0 |
| **Years 5-9** | 14.2 | 12.9 | 9.2 |
|  | | | |
| **Cohort 1 - AI** | **Reported** | **Adjusted** | **Percentage Difference** |
| **Years 0-4** | 4.5 | 4.5 | 0 |
| **Years 5-9** | 12.1 | 11.2 | 7.4 |
|  | | | |
| **Cohort 2 - TAM then AI** | **Reported** | **Adjusted** | **Percentage Difference** |
| **Years 0-4** | 5.5 | 5.5 | 0 |
| **Years 5-9** | 9.3 | 9.1 | 2.2 |
|  | | | |
| **Cohort 2 - AI** | **Reported** | **Adjusted** | **Percentage Difference** |
| **Years 0-4** | 5.1 | 5.1 | 0 |
| **Years 5-9** | 8.2 | 8.0 | 2.4 |
|  | | | |
| **Cohort 3 - TAM then AI** | **Reported** | **Adjusted** | **Percentage Difference** |
| **Years 2-4** | 4.2 | 4.1 | 2.4 |
| **Years 5-9** | 8.7 | 8.5 | 2.3 |
|  | | | |
| **Cohort 3 - TAM** | **Reported** | **Adjusted** | **Percentage Difference** |
| **Years 2-4** | 5 | 4.9 | 2.0 |
| **Years 5-9** | 10.0 | 9.5 | 5.0 |

**Paper 12: 20-year risks of breast-cancer recurrence after stopping endocrine therapy at 5 years (N Engl J Med 2017; 377: 1836-1846, DOI: 10.1056/NEJMoa1701830) NEJM**

**Distant recurrent bias**

| **N status** | **5 years** | | | **5-20 years** | | |
| --- | --- | --- | --- | --- | --- | --- |
|  | **Reported** | **Adjusted** | **Percentage Difference** | **Reported** | **Adjusted** | **Percentage Difference** |
| **N0** | 6.0 | 5.8 | 3.3 | 22.0 | 15.2 | 30.9 |
| **N1-3** | 10.0 | 9.4 | 6.0 | 31.0 | 21.2 | 31.6 |
| **N4-9** | 22.0 | 19.2 | 12.7 | 52.0 | 31.9 | 38.7 |
|  | | | | | | |
| **Cancer Mortality Bias** | | | | | | |
| **N status** | **5 years** | | | **5-20 years** | | |
|  | **Reported** | **Adjusted** | **Percentage Difference** | **Reported** | **Adjusted** | **Percentage Difference** |
| **N0** | 3.0 | 2.9 | 0 | 15.0 | 13.0 | 13.3 |
| **N1-3** | 5.0 | 5.1 | 0 | 28.0 | 24.9 | 11.1 |
| **N4-9** | 12.0 | 12.1 | 0 | 49.0 | 43.7 | 10.8 |

**Paper 14: Long-term outcomes for neoadjuvant versus adjuvant chemotherapy in early breast cancer: meta-analysis of individual patient data from ten randomised trials. EBCTCG Lancet Oncol. 2018 Jan; 19(1): 27–39. doi: 10.1016/S1470-2045(17)30777-5**

**Distant recurrent bias**

| **Neoadjuvant** | **Reported** | **Adjusted** | **Percentage Difference** |
| --- | --- | --- | --- |
| **Years 0-4** | 24.9 | 18.0 | 27.7 |
| **Years 5-9** | 33.8 | 19.9 | 41.1 |
| **Years 10-14** | 38.2 | 19.9 | 47.9 |

*Impossible to calculate the adjuvant since the data is incomplete.

**Cancer Mortality Bias**

| **Neoadjuvant** | **Reported** | **Adjusted** | **Percentage Difference** |
| --- | --- | --- | --- |
| **Years 0-4** | 18.1 | 18.3 | -1.1* |
| **Years 5-9** | 28.5 | 27.0 | 5.3 |
| **Years 10-14** | 34.4 | 29.6 | 13.9 |

| **Adjuvant** | **Reported** | **Adjusted** | **Percentage Difference** |
| --- | --- | --- | --- |
| **Years 0-4** | 16.2 | 16.4 | -1.1* |
| **Years 5-9** | 26.6 | 25.2 | 5.3 |
| **Years 10-14** | 33.7 | 29.4 | 12.8 |

**Appendix C**

**Paper 2: Effects of radiotherapy and of differences in the extent of surgery for early breast cancer on local recurrence and on 15-year survival: an overview of the randomised trials The Lancet 2005; 366: 2087-2106**

**Cancer Mortality Bias**

| **All node negative patients** | |
| --- | --- |
| **BCS +/- RT** | **Absolute Change in Rx** |
| **Years 0-4** | -0.1 |
| **Years 5-9** | 0.5 |
| **Years 10-14** | 0.2 |

| **All node positive patients** | |
| --- | --- |
| **BCS +/- RT** | **Absolute Change in Rx** |
| **Years 0-4** | 0.1 |
| **Years 5-9** | 0.8 |
| **Years 10-14** | -2.6 |

| **Mastectomy and node negative patients** | |
| --- | --- |
| **Mx +/- RT** | **Absolute Change in Rx** |
| **Years 0-4** | 0 |
| **Years 5-9** | 0.9 |
| **Years 10-14** | 0.2 |

| **Mastectomy and node positive patients** | |
| --- | --- |
| **Mx +/- RT** | **Absolute Change in Rx** |
| **Years 0-4** | 0.1 |
| **Years 5-9** | 1.1 |
| **Years 10-14** | 3.2 |

**Paper 4:** [**Meta-analysis of breast cancer outcomes in adjuvant trials of aromatase inhibitors versus tamoxifen.**](https://www.ctsu.ox.ac.uk/publications/34994) **Dowsett M. et al, (2010), J clin oncol, 28, 509 – 518**

**Cancer Mortality Bias**

| **Cohort 1 vs 2** | **Absolute Change in Rx** |
| --- | --- |
| **Tamoxifen vs AI in Cohort 1** | |
| **Years 0-4** | 0.1 |
| **Years 5-9** | 0 |
|  | |
| **Tamoxifen vs AI in Cohort 2** | |
| **Years 0-4** | 0.1 |
| **Years 5-9** | 0.1 |

**Paper 5: Overview of the Randomized Trials of Radiotherapy in Ductal Carcinoma In Situ of the Breast EBCTCG J Natl Cancer Inst Monogr. 2010 Oct; 2010(41): 162–177.**

**Local recurrent bias**

| **BCS +/- RT** | **Absolute Change in Rx** |
| --- | --- |
| **Years 0-4** | -0.7 |
| **Years 5-9** | -2.9 |

**Cancer Mortality Bias**

| **BCS +/- RT** | **Absolute Change in Rx** |
| --- | --- |
| **Years 0-4** | 0.1 |
| **Years 5-9** | -0.3 |

**Paper 6:** **Effect of radiotherapy after breast-conserving surgery on 10-year recurrence and 15-year breast cancer death: meta-analysis of individual patient data for 10 801 women in 17 randomised trials EBCTCG Lancet. 2011 Nov 12; 378(9804): 1707–1716**

**Cancer Mortality Bias**

| **+/- RT** | **Absolute Change in Rx** |
| --- | --- |
| **Years 0-4** | 0.1 |
| **Years 5-9** | 0.3 |
| **Years 10-14** | -0.1 |

**Paper 7: Relevance of breast cancer hormone receptors and other factors to the efficacy of adjuvant tamoxifen: patient-level meta-analysis of randomised trials (EBCTCG) Lancet. 2011 Aug 27; 378(9793): 771–784.**

**Cancer Mortality Bias**

| **+/- Tamoxifen** | **Absolute Change in Rx** |
| --- | --- |
| **Years 0-4** | 0 |
| **Years 5-9** | -0.3 |
| **Year >= 10** | -0.7 |

**Paper 9: Effect of radiotherapy after mastectomy and axillary surgery on 10-year recurrence and 20-year breast cancer mortality: meta-analysis of individual patient data for 8135 women in 22 randomised trials EBCTCG Lancet. 2014 Jun 20; 383(9935): 2127–2135.**

**Local recurrent bias**

| **Years** | **Absolute Change in Rx** |
| --- | --- |
| **N0 +/- RT** | |
| **Years 0-4** | 0.1 |
| **Years 5-9** | 0.2 |
|  | |
| **N1-3 +/- RT** | |
| **Years 0-4** | 3.9 |
| **Years 5-9** | 8.8 |
|  | |
| **N4+ +/- RT** | |
| **Years 0-4** | 7.2 |
| **Years 5-9** | 13.8 |

**Cancer Mortality Bias**

| **N0 +/-RT** | **Absolute Change in Rx** |  | **N1-3 +/-RT** | **Absolute Change in Rx** |  | **N4+ +/-RT** | **Absolute Change in Rx** |
| --- | --- | --- | --- | --- | --- | --- | --- |
| **Years 0-4** | 0 |  | **Years 0-4** | 0.2 |  | **Years 0-4** | -0.1 |
| **Years 5-9** | 0.5 |  | **Years 5-9** | -0.4 |  | **Years 5-9** | 1.6 |
| **Years 10-14** | -0.1 |  | **Years 10-14** | 3.1 |  | **Years 10-14** | 1.2 |
| **Years 15-19** | 3.7 |  | **Years 15-19** | -1.9 |  | **Years 15-19** | 26 |

**Paper 10: Adjuvant bisphosphonate treatment in early breast cancer: meta-analyses of individual patient data from randomised trials (Lancet 2015; 386: 1353-61)**

**Distant recurrence**

| **All Bisphosphonates** | **Absolute Change in Rx** |
| --- | --- |
| **Years 0-4** | 5.6 |
| **Years 5-9** | 0.8 |
|  | |
| **Pre- Bisph.** | **Absolute Change in Rx** |
| **Years 0-4** | 0 |
| **Years 5-9** | 0.4 |
|  | |
| **Post- Bisph.** | **Absolute Change in Rx** |
| **Years 0-4** | 0.4 |
| **Years 5-9** | 1.4 |

**Cancer Mortality Bias**

| **All Bisphosphonates** | **Absolute Change in Rx** |
| --- | --- |
| **Years 0-4** | 0 |
| **Years 5-9** | -0.1 |
|  | |
| **Pre- Bisph.** | **Absolute Change in Rx** |
| **Years 0-4** | 0 |
| **Years 5-9** | -0.1 |
|  | |
| **Post- Bisph.** | **Absolute Change in Rx** |
| **Years 0-4** | -0.1 |
| **Years 5-9** | -0.1 |

**Paper 11: Aromatase inhibitors versus tamoxifen in early breast cancer: patient-level meta-analysis of the randomised trials (Lancet 2015; 386: 1341-52)**

**Distant Recurrence**

| **Cohort 1 - TAM** | **Absolute Change in Rx** |
| --- | --- |
| **Years 0-4** | 0.3 |
| **Years 5-9** | 1.3 |
|  | |
| **Cohort 2 - TAM then AI** | **Absolute Change in Rx** |
| **Years 0-4** | 0.1 |
| **Years 5-9** | 0.2 |
|  | |
| **Cohort 3 - TAM then AI** | **Absolute Change in Rx** |
| **Years 2-4** | 0.1 |
| **Years 5-9** | 0.7 |

**Cancer Mortality Bias**

| **Cohort 1 - TAM** | **Absolute Change in Rx** |
| --- | --- |
| **Years 0-4** | 0 |
| **Years 5-9** | 0.4 |
|  | |
| **Cohort 2 - TAM then AI** | **Absolute Change in Rx** |
| **Years 0-4** | 0 |
| **Years 5-9** | 0 |
|  | |
| **Cohort 3 - TAM then AI** | **Absolute Change in Rx** |
| **Years 2-4** | 0 |
| **Years 5-9** | -0.3 |
